# Accuracy of Plain Digital Radiography for the Detection of Gastrointestinal Masses in Dogs and Cats

**DOI:** 10.3390/ani16020292

**Published:** 2026-01-17

**Authors:** Keaton Cortez, Agustina Anson, Leslie Schwarz, Nathan Biedak, Tatiana Noel, Adam South

**Affiliations:** Clinical Sciences Department, Cummings School of Veterinary Medicine, Tufts University, North Grafton, MA 01536, USAtatiana.noel@colostate.edu (T.N.);

**Keywords:** stomach, intestines, neoplasia, radiographs, dogs, cats

## Abstract

Abdominal radiography is commonly used as an initial diagnostic tool in dogs and cats with gastrointestinal (GI) signs. Historically, abdominal radiographs were considered unreliable for detecting GI masses, with detection rates below 50%. The purpose of this retrospective, case–control study was to determine the accuracy of abdominal radiographs in identifying the presence and location of GI masses and to assess the influence of the reviewer experience. Radiographs from 114 dogs and 111 cats were reviewed by two board-certified radiologists, one first year radiology resident, and one rotating intern. Patients were categorized into three groups: animals with a GI mass greater than 2 cm (dogs *n* = 44; cats *n* = 41), animals with a normal abdomen (both *n* = 50), and animals with abdominal disease but no GI mass (both *n* = 20). Reviewers demonstrated high specificity but low sensitivity for both detection and localization of GI masses. Sensitivity for detecting a mass ranged from 34 to 64% in dogs and 36 to 71% in cats; specificity exceeded 87% in dogs and 92% in cats. Sensitivity for location identification ranged from 9 to 58% in dogs and 21 to 68% in cats; specificity exceeded 76% in dogs and 81% in cats. No statistically significant differences in detection rates were found among reviewers. The accuracy of plain digital radiography for the detection of gastrointestinal masses in dogs (75%) and cats (81%) is better than previously reported film radiography but remains inferior to other imaging modalities. However, its high specificity supports its clinical utility in ruling out gastrointestinal masses.

## Simple Summary

Abdominal radiographs are commonly performed in cats and dogs when presenting with gastrointestinal signs; however, the detection of gastrointestinal masses is anecdotally unreliable. To further evaluate the efficacy of abdominal radiographs in detecting gastrointestinal masses, radiographs of 222 dogs and cats were reviewed. 85 had a gastrointestinal mass, 100 were normal, and 40 had gastrointestinal disease, but no masses. Statistical comparisons were conducted between species and between reviewers with varying levels of experience. The accuracy of radiography for the detection of gastrointestinal masses was significantly higher than previously reported. Cats had a more accurate detection rate than dogs, and there were no significant differences in accuracy between reviewers of varying experience. Although advanced imaging such as ultrasound or computed tomography remain superior, abdominal radiographs are clinically useful as a screening tool in ruling out gastrointestinal masses.

## 1. Introduction

Gastrointestinal (GI) tract neoplasia is relatively uncommon in dogs and cats, accounting for 3–10% of all tumors in these species [1]. Adenocarcinoma (AC) is the most common primary GI neoplasia in dogs, while lymphoma is the most common in cats [2,3,4]. Other malignancies include leiomyosarcoma, gastrointestinal stroma tumor (GIST), and mast cell tumors. Benign tumors have been reported in both dogs and cats and include polyps and leiomyomas.

Abdominal radiographs are often the initial imaging technique to evaluate dogs and cats with GI signs because of the low cost, wide availability, and noninvasive nature [5]. Historically, the diagnostic utility of plain radiographs for detecting GI masses has been considered limited, with anecdotally reported detection rates as low as 50% or less [6,7,8,9]. While the use of positive contrast upper GI studies can increase the sensitivity, such studies still fail to detect lesions in a significant number of cases [6]. Abdominal ultrasound is considered superior for the evaluation of morphologic lesions affecting the GI tract; however, its limitations, such as gas inhibiting complete visualization of the GI tract, necessity of an expert operator, patient size, and high equipment costs, leave abdominal radiography as the sole diagnostic imaging tool in some cases [5].

In human medicine, abdominal radiography continues to be the initial diagnostic step in patients with acute abdominal symptoms, reportedly providing a definitive diagnosis in 50–60% of patients, equivocal results in 20–30%, and normal, nonspecific, or misleading results in 10–20% [9,10]. The diagnostic accuracy of radiographs in the diagnosis of colorectal cancer has been reported as high as 84.9% [11]. When radiographs are inconclusive, additional contrast-enhanced or cross-sectional imaging modalities are often employed [12]. In contrast, veterinary practitioners often lack access to advanced diagnostic imaging techniques (e.g., computed tomography or magnetic resonance imaging), making careful case selection and optimization of radiographic technique essential. However, to the authors’ knowledge, no published studies have quantified the sensitivity or specificity of plain abdominal radiographs for this purpose.

Furthermore, the detection of GI masses on radiographs may vary with the location of the tumor, body conformation, and radiographic detail, especially in large-breed dogs or in patients with poor abdominal serosal contrast [5,13]. The experience level of the interpreter may also influence detection accuracy, but this has not been systematically evaluated in the literature.

The primary aim of this study was to determine the accuracy of plain abdominal radiographs to detect the presence of GI masses in cats and dogs. A secondary goal was to assess the impact of reviewer experience on detection performance. We hypothesized the following: (1) the detection rate of GI masses on abdominal radiographs would be higher than anecdotally reported, (2) the detection rate of GI masses in cats would be higher than in dogs due to a better and more predictable serosal detail in the abdomen, and (3) diagnostic performance would be highest for board-certified radiologists as compared to a first-year radiology resident and a rotating intern.

## 2. Materials and Methods

This was a retrospective, case–control study using three populations of both dogs and cats. The first population (group 1) was selected based on the confirmation of a GI mass greater than 2 cm [13] using ultrasound, computed tomography, surgery, and/or necropsy. Identification of these cases was conducted via search of the Foster Hospital for Small Animals medical record database, Diagnostic Imaging database, and Pathology database combining the terms “gastric, duodenal, jejunal, ileal, cecal, colonic or intestinal” and “mass, neoplasia, carcinoma, leiomyoma, leiomyosarcoma, lymphoma and spindle cell neoplasia.” The second population (group 2) was the control and was selected based on both abdominal radiographs and ultrasound that were interpreted as normal. Identification of these cases was conducted via search of the Foster Hospital for Small Animals medical record database and Diagnostic Imaging database for term “normal/unremarkable abdomen”. The third population (group 3) included animals with a variety of GI diseases but no masses. Identification of these cases was conducted via search of the Foster Hospital for Small Animals medical record database and Diagnostic Imaging database for terms “foreign body, intussusception, gastritis, enteritis, enteropathy, colitis, pancreatitis.”

For all groups, at least two orthogonal abdominal radiographs were required. Descriptive data collected for each patient included breed, age, sex, weight, and reproductive status (intact or neutered/spayed).

The abdominal radiographs of these populations were anonymized, randomized, and reviewed independently by two board-certified radiologists (AA, LS), one first-year radiology resident (NB), and one rotating intern (TN). Reviewers were blinded to group assignments and patient history.

Each reviewer evaluated the images to determine (1) whether a GI mass was present, and (2) if present, the suspected anatomical origin (stomach, duodenum, jejunum, ileum, ileocecocolic junction [ICJ], colon). Additional radiographic features evaluated included margination and size of the mass, serosal detail, presence of mineralization within the mass, gravel sign, mass effect, free gas, and mechanical ileus. The term gravel sign was used to describe any accumulation of fine, radiopaque particulate material within the intestinal tract. A mechanical ileus was considered when two populations of intestinal loops were present.

### Statistical Analysis

The accuracy, sensitivity, specificity, false positive, false negative, and positive/negative predictive values (PPV/NPV) of radiographs in detecting GI masses were calculated. The second portion of the statistical analysis was to examine the potential increase in diagnostic accuracy in radiologists as compared to less experienced reviewers. There was computation of the inter-rater reliability to assess the agreement between the radiologists and less experienced reviewers. To compute the inter-rater reliability, Cohen’s kappa was used where values between 0.75 and 1.0 were excellent, 0.60 and 0.75 were considered good, 0.4 and 0.60 were considered fair, and below 0.4 was considered poor [14]. All analyses were based on the data from the board-certified radiologists, regardless of the results of inter-rater reliability. All analyses were stratified by species (cats, dogs), weight (1–10 kg, 10–25 kg, >25 kg), and location (stomach, duodenum, jejunum, ileum, ICJ, and colon). All analyses were conducted using SPSS (version 27.0, SPSS Inc., Chicago, IL, USA) and MedCalc (version 19.4, MedCalc Software, Ostend, Belgium).

## 3. Results

Medical records of approximately 500 dogs and cats with abdominal radiographs were evaluated. Exclusion from the study was most commonly due to lack of orthogonal radiographs, a GI mass less than 2 cm, or lack of advanced imaging.

### 3.1. Dogs

A total of 114 dogs was included, 44 dogs with a gastrointestinal mass (group 1), 50 dogs with a normal abdomen (group 2), and 20 dogs with abdominal disease but no GI mass (group 3). There were 20 males (19 neutered, 1 intact) and 24 females (20 neutered, 4 intact) in group 1, 21 males (14 neutered, 7 intact) and 29 females (27 neutered, 2 intact) in group 2, and 10 males (9 neutered, 1 intact) and 10 females (all neutered) in group 3. The age ranges for each group were 1–18 years, 5 months–14 years, and 6 months–15 years, respectively.

Histopathologic diagnoses were available for 22 of the 44 dogs with a GI mass. Among these, 13 were adenocarcinomas, 3 leiomyomas, 3 lymphomas, and 1 each of angioleiomyosarcoma, gastrointestinal stromal cell tumor (GIST), and undifferentiated sarcoma. The origin of the mass was available for all 44 dogs, including 12 gastric, 27 small intestinal (8 duodenal, 18 jejunal, 1 ileal), and 5 large intestinal (3 ICJ, 2 colonic). Of the 13 adenocarcinomas, 1 originated from the stomach, 9 from the small bowel, and 3 from the large bowel. All three leiomyomas were gastric in origin. The three lymphomas were located 1 in the small bowel and 2 in the large bowel. The angioleiomyosarcoma and GIST originated from the stomach while the sarcoma was small intestinal in origin.

In dogs with GI masses (group 1), steatitis was the most common concurrent ultrasonographic abnormality (25/44 [57%]), followed by mechanical ileus (11/44 [25%]), gastrointestinal foreign material (6/44 [14%]), peritoneal effusion (5/44 [11%]), pneumoperitoneum (4/44 [9%]), mineralization within the mass (3/44 [7%]), and gravel sign (3/44 [7%]).

In group 3, enteritis was the most frequent diagnosis (11/20 [55%]), followed by mechanical ileus (4/20 [20%]), colitis (2/20 [10%]), pancreatitis (1/20 [5%]), lymphangiectasia (1/20 [5%]), and gastric ulceration (1/20 [5%]).

### 3.2. Cats

A total of 111 cats was included, 41 cats with a gastrointestinal mass (group 1), 50 cats with a normal abdomen (group 2), and 20 cats with abdominal disease but no GI mass (group 3). Group 1 included 22 males (all neutered) and 19 females (all neutered), group 2 included 22 males (21 neutered, 1 intact) and 28 females (26 neutered, 2 intact), and group 3 included 6 males (all neutered) and 14 females (13 neutered, 1 intact). The age ranges for each group were 1.5–19 years, 5 months–18 years, and 8 months–16 years, respectively.

Histopathologic diagnoses were available for 31 of the 41 cats with a GI mass including 14 lymphomas, 10 adenocarcinomas, 3 adenomatous polyps, 2 mast cell tumors, and 2 sarcomas. The origin of the gastrointestinal mass was available in all cases and included 8 gastric, 17 small intestinal (2 duodenal, 15 jejunal), and 16 large intestinal masses (6 ICJ, 2 cecal, 8 colonic). Of the 14 lymphomas, 4 were gastric, 6 small intestinal, and 4 large intestinal in origin. Of the adenocarcinomas, four arose from the small bowel and six from the large bowel. Two of the adenomatous polyps originated from the stomach and one from the small bowel. The two mast cell tumors originated from the small and large bowel, and the two sarcomas from the large bowel.

Among cats in group 1, steatitis was the most common concurrent ultrasonographic finding (24/41 [59%]), followed by mechanical ileus (11/41 [27%]), intra-luminal foreign material (9/41 [22%]), and peritoneal effusion (6/41 [15%]). Mineralization within the mass, pneumoperitoneum, and gravel sign were each seen in 2 of the 41 cases (5%).

The most common abdominal diseases in group 3 were enteritis (8/20 [40%], followed by mechanical ileus (7/20 [35%]), pancreatitis (3/20 [15%]), and one each of colitis and constipation (2/20 [10%]).

### 3.3. Radiographic Evaluation

The sensitivity, specificity, PPV, NPV, and accuracy of each reviewer for identification of a mass within the GI tract and location of the mass are summarized in Table 1. All reviewers demonstrated high specificity for detection of a mass in the gastrointestinal tract in both cats (>92%) and dogs (>87%). However, sensitivity was lower across all reviewers, ranging from 36% to 71% in cats and from 34% to 64% in dogs. The mean accuracy for detecting a GI mass was 80.6% in cats (range 73.9–88.3%) and 75.2% in dogs (range 66.7–85.1%).

Performance in identifying the location of the mass was similarly characterized by high specificity and low sensitivity. In cats, specificity exceeded 81% and sensitivity ranged from 21% to 68%, with a mean accuracy of 74.1% (range 66.7–85.6%). In dogs, specificity exceeded 76%, and sensitivity ranged from 9% to 58%, with a mean accuracy of 68.4% (range 56.1–79.82%).

There was no statistical significance in inter-rater reliability between the board-certified radiologists, the first-year radiology resident, and the rotating intern. Four dogs in group 2 were incorrectly identified as having a GI mass, three by the first-year radiology resident and two by the rotating intern, with one of these cases misclassified by both. In these cases, the stomach was the most common incorrectly identified location. No cats in group 2 were incorrectly identified as having a gastrointestinal mass.

**Figure 1:**
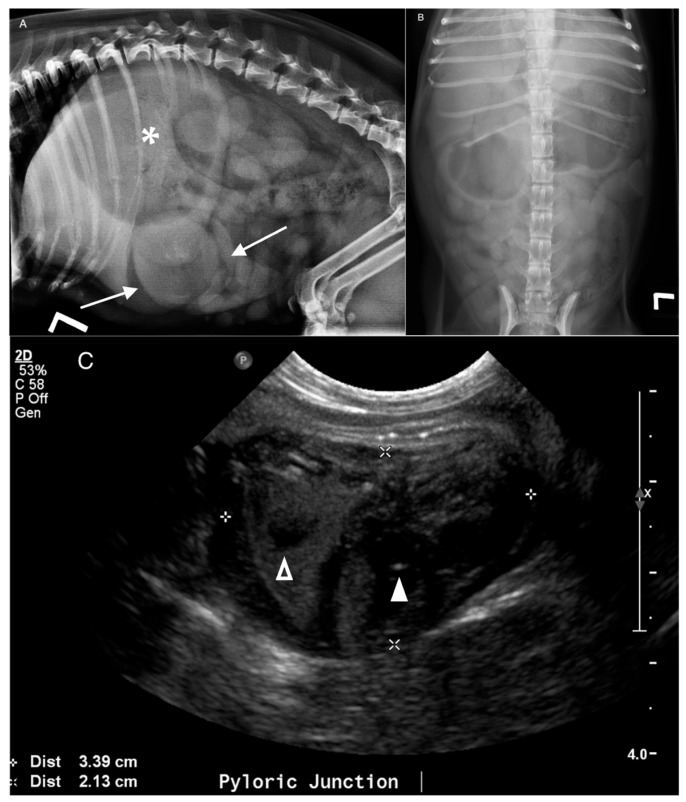
A 15-year-old spayed female Chihuahua presented for chronic progressive vomiting. (**A**) Lateral and (**B**) ventrodorsal abdominal radiographs. Note the moderate distension of the stomach (*) with gas and fluid with a rounded soft tissue opacity (arrows) and faint stippled mineral. (**C**) Corresponding ultrasonographic image. Large, ovoid, heterogeneously hypoechoic mass (calipers) in the dorsal wall of the pylorus with small cavitations (hollow arrowhead) and mineral foci (arrowhead). All reviewers correctly identified the mass and location. Histopathology: Angioleiomyosarcoma.

**Figure 2:**
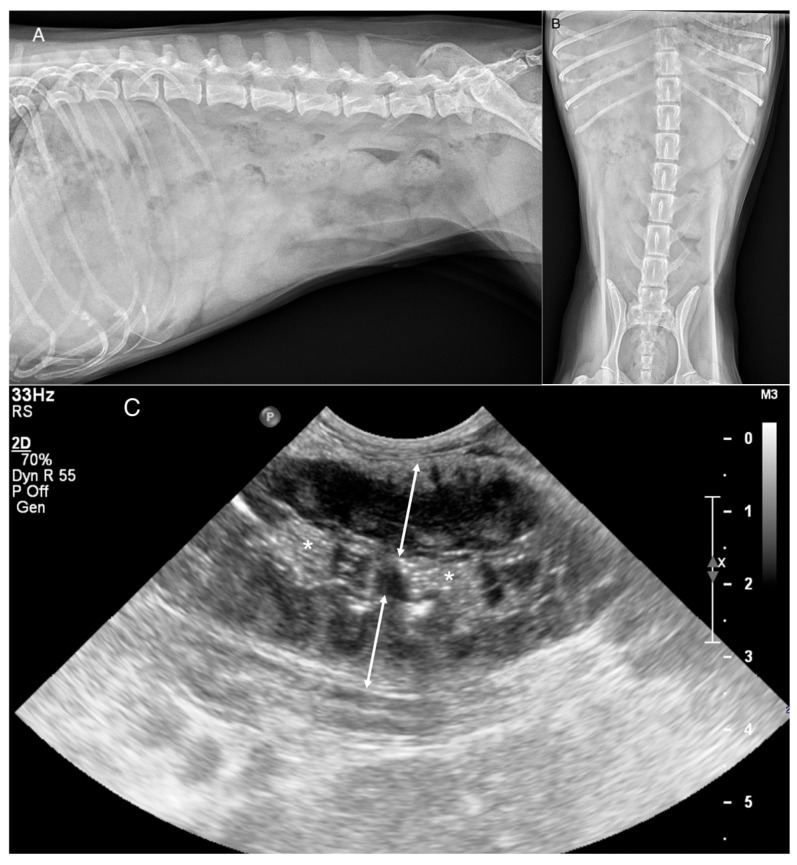
A 7-year-old spayed female Yorkshire Terrier presented for chronic vomiting and weight loss. (**A**) Lateral and (**B**) ventrodorsal abdominal radiographs. Note the fluid-filled small intestines and decreased peritoneal serosal detail. A mass was not visualized in this case. (**C**) Corresponding ultrasonographic image. Circumferential heterogeneously hypoechoic thickening affecting a long segment of jejunum (double headed arrows). All reviewers classified the case as no mass present. * = lumen. Histopathology: Lymphoma.

Among the 44 dogs in group 1, seven masses (16%) were correctly identified by all reviewers. Of these, five were small intestinal and two were gastric in origin (Figure 1). In contrast, five cases (11%) were incorrectly labeled as “no mass present” by all reviewers, four small intestinal origin and one gastric origin (Figure 2). Three of the five cases (two small bowel, one gastric) had no additional abdominal changes while the remaining two cases had steatitis, peritoneal effusion, or both. Mechanical obstruction was identified unanimously in seven cases (16%), all corresponding to carcinomas. Of these, five cases had a concurrent gravel sign.

**Figure 3:**
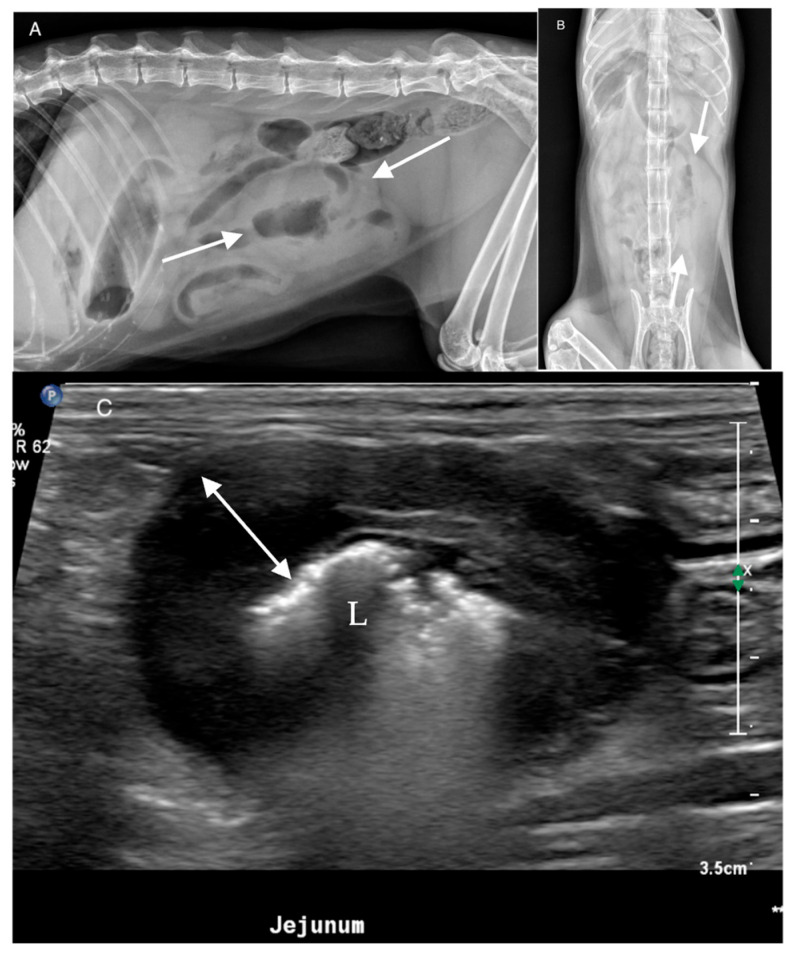
A 12-year-old spayed female Domestic Shorthair presented chronic progressive hyporexia and weight loss. (**A**) Lateral and (**B**) ventrodorsal abdominal radiograph. Ill-defined increased soft tissue in the mid caudal abdomen creating a mass-like appearance (arrows) with superimposed tubular gas and small gas foci. (**C**) Corresponding ultrasonographic image. Severe circumferential hypoechoic thickening of the jejunal wall with loss of layering (double headed arrow). All reviewers correctly identified the mass and location. L = lumen. Histopathology: Large-cell lymphoma.

**Figure 4:**
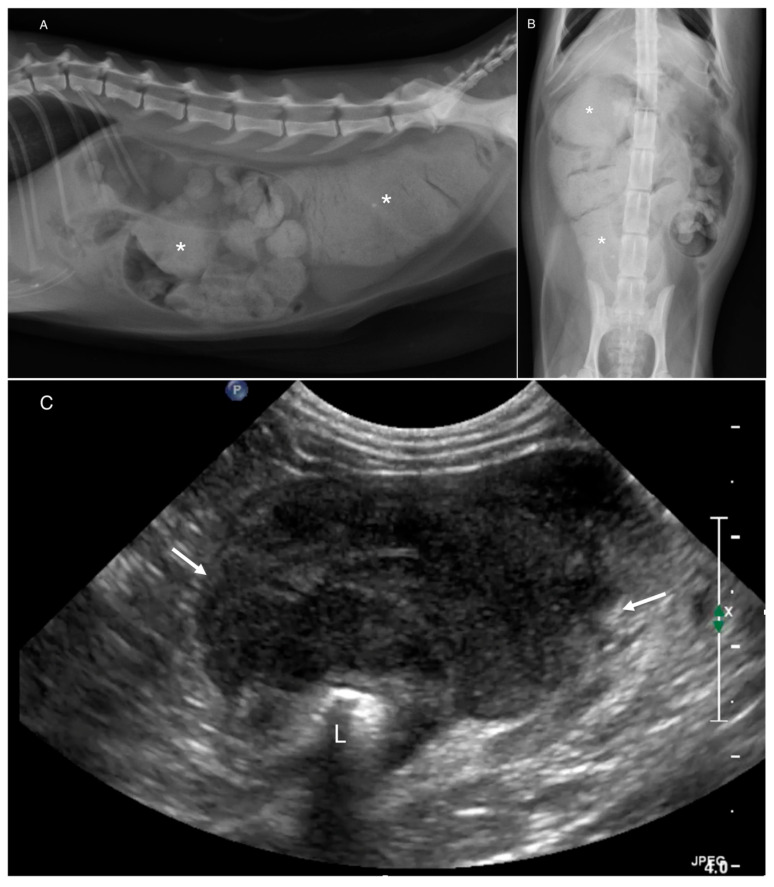
A 12-year-old spayed female Domestic Shorthair presented for constipation. (**A**) Lateral and (**B**) ventrodorsal abdominal radiographs. Large volume of desiccated fecal material (*) in the distal descending colon. (**C**) Corresponding ultrasonographic image. Amorphous, lobular, heterogeneously hypoechoic mass (arrows) centered at the ileocolic junction. L = lumen. All reviewers identified as no mass present. Histopathology: Large-cell lymphoma.

Among the 41 cats in group 1, nine masses (21%) were correctly identified by all reviewers. Of these, six were small intestinal, two large intestinal, and one gastric in origin (Figure 3). Four cases (10%) were missed by all reviewers, including two large intestinal and one small intestinal in origin (Figure 4). Two of the four missed cases, both large intestinal in origin, had no additional abdominal changes. The remaining large intestinal mass caused steatitis and free peritoneal gas while the small intestinal mass had concurrent steatitis. A gravel sign was identified unanimously in three cases (7%), all corresponding to carcinomas. Of these, two cases had a concurrent mechanical obstruction.

## 4. Discussion

This study provides valuable insight into the diagnostic capability of digital radiography for identifying gastrointestinal masses in dogs and cats and represents the first objective quantification of its accuracy. Historically, the utility of abdominal radiographs for detecting GI masses has been considered limited, with anecdotal detection rates at 50% or lower [6,7,8,9]. The present findings demonstrate a measurable improvement in diagnostic accuracy compared to historical reports, likely reflecting the advantages of digital imaging systems over traditional film radiography.

The results of this study demonstrated that while the sensitivity of radiographs for detecting GI masses in dogs ranged from 34% to 64% and in cats from 36 to 71%, the specificity was high, exceeding 87% in dogs and 92% in cats. This suggests that when an abdominal radiograph is reviewed and deemed normal, there is a strong likelihood that no mass is present. The accuracy rates of 75% for dogs and 81% for cats are significant improvements over previously reported anecdotal rates for film radiography. The implementation of digital radiography in veterinary medicine has provided the opportunity to improve interpretation efficiency and minimize the possibility of missed lesions. Postprocessing algorithms for viewing digital radiographs allow the reviewer to manipulate an image to individual preferences. The ability to manipulate image contrast and brightness in digital radiography compared to film radiography may contribute to this increase in diagnostic accuracy [15].

Radiographic detection accuracy was consistently higher in cats than in dogs, supporting our initial hypothesis. The feline abdomen typically contains greater peritoneal fat relative to size and less soft tissue superimposition, providing superior serosal detail and visualization of organ boundaries [13,16]. In contrast, large or deep-chested dogs present challenges such as increased summation and reduced contrast due to body conformation that may obscure subtle lesions. This difference emphasizes the importance of optimizing radiographic technique and that positioning is essential in large dogs, where gastrointestinal masses are more easily missed.

Localization of GI masses proved substantially more difficult than detection, with sensitivity rates ranging from 9 to 58% in dogs and 21 to 68% in cats. This result underscores the inherent limitations of radiographic interpretation, as accurate anatomical localization is frequently compromised by summation and superimposition of abdominal viscera [13]. Importantly, while imprecise localization may limit the ability to define the exact site of origin and therefore primarily impacts surgical planning, it does not necessarily preclude appropriate clinical decision-making. In many cases, radiographic detection of a suspected mass alone is sufficient to prompt further diagnostic evaluation with advanced imaging modalities, such as ultrasonography or computed tomography, which are better suited for precise localization and characterization [2,5,6].

Reviewers were most accurate with canine gastric masses; however, gastric origin was also the most incorrectly identified in the control groups. Evaluation of the stomach can vary based on patient positioning and luminal contents. Luminal gas can serve as a negative contrast agent which increases the conspicuity of mural lesions, increasing the accuracy of detection. Conversely, luminal fluid silhouetting asymmetrically with the gastric wall can create artifactual thickening, which can be misidentified as a mass while food within the stomach may obscure the lesion [5,13]. These changes, present in this study, likely lead to the dichotomy in evaluation of the stomach.

The distribution of tumor types and locations in this study aligns with previous reports: adenocarcinoma predominated in dogs and lymphoma in cats [2,3,4]. In dogs, adenocarcinomas were predominately of small intestinal origin, consistent with earlier studies describing the jejunum and ileum as the most common sites of intestinal epithelial malignancy [2,17]. In contrast, mesenchymal tumors (leiomyomas, leiomyosarcoma, and GIST) originated from the stomach. Colon and cecum have been described as the most common localization of GIST, whereas the stomach is more often affected by leiomyomas and leiomyosarcomas [18].

In cats, lymphomas and adenocarcinomas were common in both small and large intestines. Feline intestinal lymphoma most frequently affects the jejunum, whereas adenocarcinoma is overrepresented in the ileocecocolic junction and colon [3,4].

The presence of mechanical obstruction and/or a gravel sign was associated exclusively with intestinal adenocarcinomas in our study. Intestinal adenocarcinomas are often described as annular (“napkin-ring”) lesions that constrict the lumen and cause chronic partial obstruction [13,19,20]. As intestinal contents stagnate proximal to the lesion, fine mineralized particulate material accumulates, producing the characteristic “gravel sign”—a radiopaque pattern regarded as a hallmark of chronic mechanical obstruction of neoplastic origin [20].

Contrary to our third hypothesis, no significant difference in diagnostic accuracy was found among reviewers of varying experiences. This finding further supports that the difficulty of identifying gastrointestinal masses lies more in the inherent limitations of the modality than in observer expertise and suggests that even experienced radiologists may encounter difficulties in identifying GI masses.

This study also has inherent bias as the reviewers were trying to identify GI masses without signalment or history of the patient. It is possible that whenever a mass effect, effusion, or mechanical obstruction were present, the reviewers were more inclined to state that there was a GI mass. The addition of a second control group of animals with abdominal disease, but no GI mass, likely reduced this bias but did not completely eliminate it. Reviewing in this way is dissimilar to the normal practice of a radiologist, which almost always includes signalment and history. A future study may blind the reviewers to the disease process they are evaluating for while including signalment and history of a patient, which lends itself to real life comparison.

The clinical implications of this study are multifaceted. While plain digital radiography demonstrates utility in ruling out GI masses due to the high specificity, the low sensitivity necessitates a careful consideration of additional modalities, especially in cases where clinical suspicion remains high. The limitations of radiography such as superimposition or concurrent abdominal disease increase the need for complementary imaging modalities, namely ultrasound or computed tomography, to provide a more comprehensive evaluation of the GI tract.

The results of this study highlight the need for further research to explore factors influencing the detection of GI masses on radiographs. Variables such as body conformation or weight, size and location of the mass, and presence of other abdominal disease can all impact the interpretability of radiographic images. The retrospective nature of this study limited case selection with many of the feline masses being longer in length than thickness. Masses that tend to grow large, such as mesenchymal tumors, are more easily identified as they commonly cause regional mass effect whereas small focal masses (carcinomas) or longer affected segments (lymphoma) may be more difficult as they superimposed with surrounding intestinal loops or concurrent disease [21,22]. Future studies evaluating the accuracy of detection for specific tumor types or specific locations may further separate when radiographs are useful as a screening modality.

## 5. Conclusions

This study elucidates the challenges and strengths associated with the use of plain digital radiography for the detection of GI masses in dogs and cats. While the low sensitivity indicates that reliance on this modality may lead to missed diagnoses, the high specificity supports the continued relevance in clinical veterinary practice as a first step diagnostic. An understanding of these limitations, combined with clinical judgment, will empower veterinary practitioners to optimize the diagnostic workflow and prioritization for patients with suspected GI neoplasia.

## Figures and Tables

**Table 1:** Diagnostic performance of observers for detection and localization of gastrointestinal (GIT) masses in feline and canine patients.

Species	Observer	Evaluation	Sensitivity	Specificity	PPV	NPV	Accuracy
Feline	1 (AA)	Mass in GIT	70.7	98.6	96.7	85.2	88.3
		Location	68.4	94.5	86.7	85.2	85.6
	2 (LS)	Mass in GIT	46.3	92.9	79.2	74.7	75.7
		Location	37.1	85.5	54.2	74.7	70.3
	3 (NB)	Mass in GIT	65.9	95.7	90.0	82.7	84.7
		Location	51.7	81.7	50.0	82.7	73.9
	4 (TN)	Mass in GIT	36.6	95.7	83.3	72.0	73.9
		Location	21.2	85.9	38.9	72.0	66.7
Canine	1 (AA)	Mass in GIT	63.6	98.6	96.6	81.2	85.1
		Location	57.9	90.8	75.9	81.2	79.8
	2 (LS)	Mass in GIT	36.4	97.1	88.9	70.8	73.7
		Location	24.3	88.3	50.0	70.8	67.5
	3 (NB)	Mass in GIT	50.0	91.4	78.6	74.4	75.4
		Location	42.1	84.2	57.1	74.4	70.2
	4 (TN)	Mass in GIT	34.1	87.1	62.5	67.8	66.7
		Location	8.8	76.3	13.6	66.3	56.1

## Data Availability

The original contributions presented in this study are included in the article. Further inquiries can be directed to the corresponding author.

## Abbreviations

The following abbreviations are used in this manuscript:

GI	Gastrointestinal
AC	Adenocarcinoma
GIST	Gastrointestinal stromal tumor
ICJ	Ileocecocolic junction
PPV	Positive predictive value
NPV	Negative predictive value
